# Factors associated with mortality among critically ill pediatric patients referred to a tertiary hospital in Indonesia

**DOI:** 10.1371/journal.pone.0361504

**Published:** 2026-10-27

**Authors:** Desy Rusmawatiningtyas, Indah Kartika Murni, Dwi Astuti Dharma Putri, Antonius H. Pudjiadi, Hennie Knoester, Job B.M. van Woensel

**Affiliations:** 1 Department of Child Health, Faculty of Medicine, Public Health, and Nursing, Universitas Gadjah Mada/DR Sardjito General Hospital, Yogyakarta, Indonesia; 2 Department of Child Health, Faculty of Medicine, Universitas Indonesia, Jakarta, Indonesia; 3 Department of Pediatric Intensive Care, Emma Children’s Hospital/Amsterdam University Medical Centers, University of Amsterdam, Amsterdam, The Netherlands; Siddhi Memorial Hospital, NEPAL

## Abstract

**Background:**

An efficient referral system is an important but often under-recognized determinant of survival in critically ill children. Previous studies have reported high mortality rates among pediatric patients transferred to tertiary care facilities. This study aimed to identify factors associated with Pediatric Intensive Care Unit (PICU) mortality among critically ill children transferred and admitted to a tertiary-level referral hospital in Yogyakarta, Indonesia.

**Material and methods:**

This prospective cohort study was conducted over a 12-month period (July 1, 2023, to June 30, 2024) and included all critically ill children admitted to the PICU following interhospital referral. Demographic, clinical, referral, and transport data were prospectively collected. Univariable and multivariable logistic regression analyses were performed to identify factors independently associated with PICU mortality.

**Results:**

A total of 334 children were included, with a median age of 6 years. PICU mortality was 25.4%. The most common referral indication based on the Pediatric Assessment Triangle (PAT) was central nervous system dysfunction. Pre-referral management most commonly included oxygen therapy, antibiotic administration, and fluid resuscitation. Compared with survivors, non-survivors had higher PELOD-2 scores and were more likely to require intubation, mechanical ventilation, and vasoactive support before referral. In the multivariable analysis, pre-referral vasoactive support (adjusted OR 2.23, 95% CI 1.20–4.15; P = 0.011) and higher PELOD-2 scores within the first 24 hours after PICU admission (adjusted OR 1.32 per 1-point increase, 95% CI 1.20–1.45; P < 0.001) were independently associated with PICU mortality. Referral distance, decision time, and transfer time were not independently associated with mortality.

**Conclusion:**

PICU mortality among critically ill children transferred and admitted to a tertiary-care hospital in Indonesia remains high. Pre-referral vasoactive support and higher PELOD-2 scores at PICU admission were independently associated with PICU mortality. These findings generate the hypothesis that improvements in early stabilization and communication may improve outcomes, warranting prospective evaluation.

## Introduction

Low- and middle-income countries (LMICs), including Indonesia, continue to face significant challenges in pediatric healthcare, particularly in reducing childhood mortality [1,2]. One of the main elements of primary health care is the referral system, which allows patients to obtain services at health centers with superior facilities and infrastructure. Indonesia still faces the challenge of inadequate establishment of well-organized referral systems to address the needs of critically ill children. Specialized facilities such as tertiary-level Pediatric Intensive Care Units (PICU) are sparse, and previous studies indicate that mortality remains high among critically ill children referred to a tertiary PICU [3]. Pediatric intensive care services in Indonesia are organized into primary, secondary, and tertiary levels according to service capability. The national Hospital Information System (SIRS) records PICU capacity at individual hospitals, including PICU beds with ventilators; however, publicly available national aggregate data on the number of functioning PICUs, their level of service, or PICU beds per population remain limited.

General practitioners (GPs) with experience in recognizing alarm signs in critically ill children, as well as an efficient pre-referral communication system for critically ill children, are crucial for making optimal use of these limited resources. It has been shown that many GPs expressed limited confidence in recognizing and managing pediatric emergencies in pre-referral settings [4–7]. Yet, the importance of these determinants for outcomes in critically ill children remains less well understood [2,8–13]. While most LMICs have formal or informal referral systems in place within their tiered healthcare structures; these systems frequently encounter operational, technical, and logistical difficulties, which hinder timely, efficient, and appropriate referral [2,9–11,14].

In terms of communication between healthcare facilities, Indonesia has implemented the Integrated Referral System (*Sistem Informasi Rujukan Terintegrasi*, SISRUTE). This is an Information and Technology (IT)-based platform that supports communication between pre-referral and referral healthcare services through various channels, including Short Message Service (SMS), Android applications, and web-based platforms. The system is designed to enable prompt information exchange with the aim of improving patient safety, quality of care, and satisfaction [3,14,15]. However, this system has several limitations in facilitating pre-referral communication in order to refer patients with critical illnesses [3].

One study identified several challenges in different stages of the patient’s journey—from the community level to the tertiary care facility in our setting [3]. Numerous studies have shown that the outcome of a critically ill referral patient is affected by (1) whether the child had timely access to a healthcare provider during the early phase of disease, (2) whether the initial provider was able to recognize alarm symptoms and respond appropriately, (3) whether communication between facilities was feasible, and (4) whether the referring hospital was supported by a transport team in order to timely and effectively initiate referral to a higher-level facility [2,9–13,16]. Considering Indonesia’s unique characteristics, including its vast territory, archipelagic nature, and variably high population density, with a limited and uneven number of PICU facilities, there is no available data on this issue. In order to improve patients’ outcomes, the aim of our study is to evaluate the factors associated with mortality among critically ill pediatric patients transferred and admitted to a tertiary-level referral hospital in Yogyakarta, Indonesia.

## Materials and methods

### Study settings and population

This prospective cohort study was conducted in the PICU at Sardjito General Hospital, Yogyakarta. As the only tertiary, public university-based hospital in Yogyakarta Province, Indonesia, our hospital serves as a major referral center for critically ill children. The majority of our pediatric referral cases originate from within Yogyakarta Province and nearby provinces such as Central Java and East Java. The nearest alternative tertiary-level PICU is located approximately 80 kilometers away, in Central Java. Our PICU is equipped with 22 beds for both medical and surgical intensive care and manages approximately 1,300 admissions annually. Half of our admissions involve pediatric patients referred from lower-level healthcare facilities [3].

### Patients and operational definition

We enrolled all pediatric patients aged 1 month to 18 years who were transferred as emergency referrals from lower-level hospitals and admitted to our PICU during the one-year study period, from July 1^st^, 2023, to June 30^th^, 2024. We defined emergency referrals as all patients referred with one of the emergency assessments based on Pediatric Assessment Triangle (PAT) by the referring hospital such as respiratory distress, respiratory failure, shock, central nervous system (CNS) dysfunction, metabolic disturbance, and cardiopulmonary failure [17]. The primary outcome was PICU mortality, defined as death occurring during PICU admission among children successfully admitted to the PICU following interhospital referral. Only children who completed interhospital transfer and were admitted to the PICU were eligible for inclusion in the study. Patients who did not reach our PICU or had insufficient clinical information were excluded because the primary outcome and key predictor variables could not be reliably assessed.

All eligible consecutive patients admitted during the study period were included in this prospective cohort study. Because this was an observational prognostic study, the sample size was determined by the number of eligible patients presenting during the pre-defined 12-month study period rather than by a formal hypothesis-testing sample size calculation. The number of outcome events was subsequently evaluated to assess the feasibility of multivariable logistic regression analysis.

We defined “Decision Time” as the interval between the referral request—sent via SISRUTE or short message service (SMS) by the referring hospitals—and the decision to accept the patient, following consultation with the attending PICU physician. “Transfer time” was defined as the interval between the time of the acceptance decision and the patient’s arrival at our Emergency Department (ED). Referral distance was defined as the estimated road distance (in kilometers) between the referring hospital and Dr. Sardjito Hospital. Distance was determined using Google Maps and analysed as a continuous variable. Effect estimates are reported per 10-km increase in referral distance. We defined the need for therapies, including antibiotics and vasoactive drugs provided at the referring hospital at the discretion of the responsible referring physicians were recorded. We defined “Length of Stay (LOS) at the referring hospital” as the length of stay reported by the referring hospital before the patient is referred to our hospital. The Pediatric Logistic Organ Dysfunction −2 (PELOD-2) score was calculated based on the applicable variables during the first 24 hours after our PICU admission [18]. Nutritional status was assessed according to the hospital pediatric nutritional assessment guideline using age-appropriate growth charts. WHO growth standards/references were used for the general pediatric population, while condition-specific growth charts were used when appropriate, such as Down syndrome growth charts for children with Down syndrome. For the present analysis, malnutrition included both undernutrition and severe malnutrition.

All patients referred to our hospital are first presented to the ED, including referrals who require PICU. In conditions where a system error occurred on the SISRUTE, all referrals for critically ill children were made via direct phone contacts to our hospital. In such cases, key time-related data, including decision time and transfer time, were obtained from communication logs between the referring hospital and our team, including phone calls and text messages.

### Data collection

Data were collected using a structured Case Record Form (CRF) and commenced immediately at the time of first contact with the referring hospital. Information gathered for each referral case included age, sex, nutritional state, distance to our hospital, referring hospital emergency assessment, location of care at referring hospital, length of stay at referring hospital, and treatment received in the referring hospital (conventional oxygen therapy, intubation, mechanical ventilation, fluid resuscitation, vasoactive drugs, and antibiotic treatment). We also record the initial data at receiving hospital, including the initial PAT at our ED, decision time, and transfer time.

Following admission to the PICU at Sardjito Hospital, additional data were recorded, including demographic information, PICU LOS, total hospital LOS, and PELOD-2 scores within the first 24 hours of PICU admission. All data were accessed for research purposes between 1 July 2023 and 30 June 2024.

### Statistical analysis

Continuous variables were summarized as mean ± standard deviation or median (interquartile range), according to their distribution, whereas categorical variables were presented as frequencies and percentages. Comparisons between survivors and non-survivors were performed using the independent t-test or Mann–Whitney U-test for continuous variables and the chi-square test or Fisher’s exact test for categorical variables, as appropriate.

Univariable logistic regression analysis was initially performed to evaluate the association between each candidate predictor and PICU mortality. Continuous variables, including age, referral distance, decision time, transfer time, and PELOD-2 score, were analyzed as continuous variables and were not categorized in the primary analysis to preserve statistical information and avoid bias associated with data-driven cut-points.

Candidate variables for multivariable logistic regression were selected a priori based on clinical relevance and existing evidence, while the results of the univariable analyses were used to support model building rather than serving as the sole criterion for variable selection. The number of variables entered into the final model was limited to reduce the risk of overfitting. Prior to multivariable analysis, multicollinearity among candidate predictors was assessed using variance inflation factors (VIF), with VIF < 5 considered acceptable.

Odds ratios (ORs) with 95% confidence intervals (95% CIs) were reported. For continuous predictors, ORs were expressed per clinically meaningful unit increase (for example, per 10-km increase in referral distance and per one-point increase in PELOD-2 score). Statistical significance was defined as p < 0.05.

### Ethical approval

Ethical approval was obtained from the Medical and Health Research Ethic Committee (MHREC) Universitas Gadjah Mada Yogyakarta, Indonesia, with reference number KE/FK/0882/EC/2022. Confidentiality and patient privacy were strictly maintained. Access to identifiable data was limited to the principal investigator and designated research assistants. All study procedures were in accordance with relevant guidelines and regulations.

## Results

During the one-year study period, our Emergency Department (ED) received 2,376 pediatric referral requests, of which 1,306 (54.9%) were considered by the referring hospitals to require PICU admission. Among these, 1,085 (83.1%) referral requests were accepted by the pediatric intensivist, and 651 (60.0%) children subsequently presented to the ED. Overall, 334 (30.8%) children were admitted to the PICU. Eleven children died in the ED before PICU admission, whereas 306 were admitted to the general pediatric ward because they no longer met the criteria for PICU admission (Fig 1).

**Fig 1:**
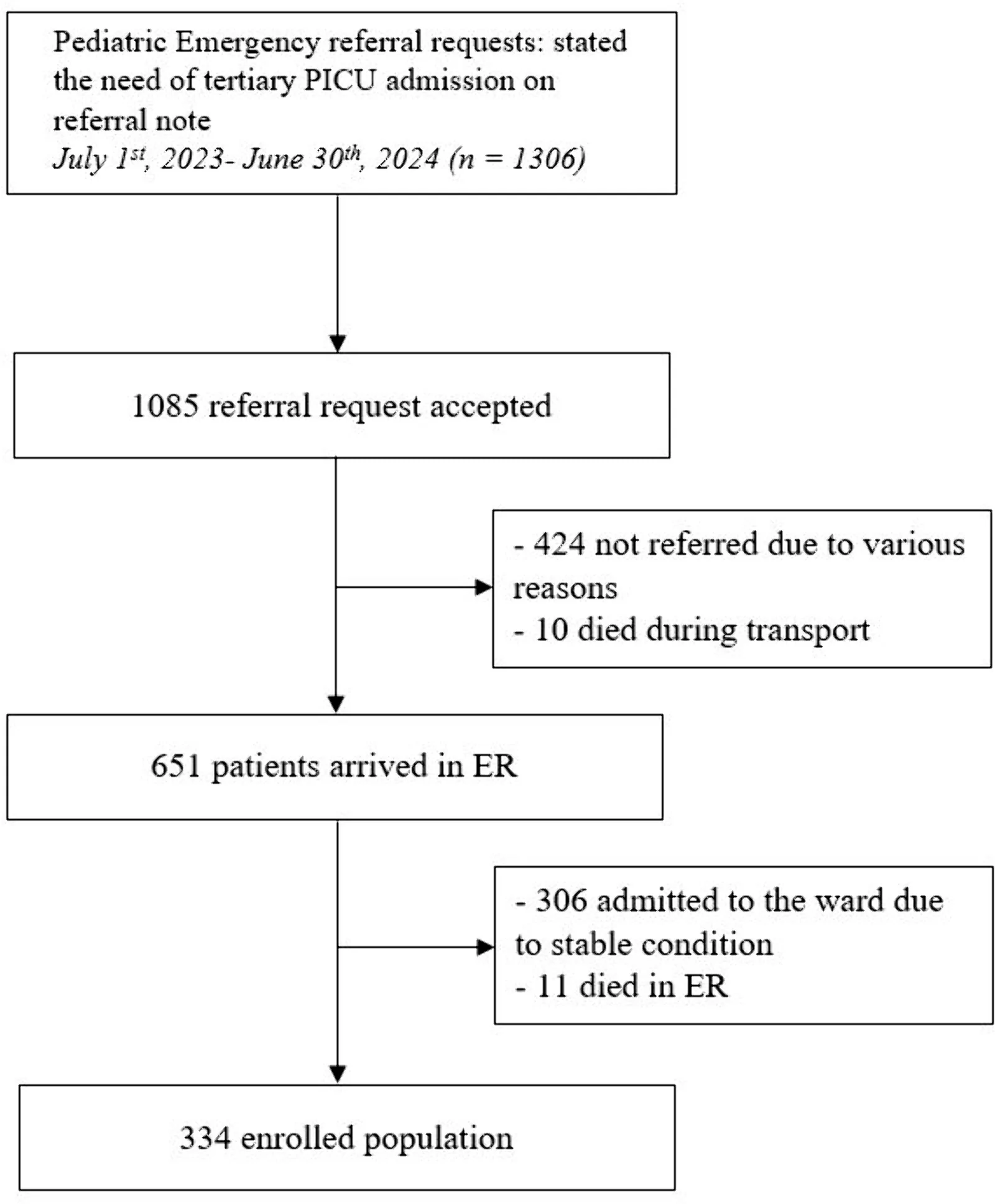
Flow Diagram of enrolled population.

A total of 434 children who had been accepted for referral did not arrive at our hospital. Ten children died during transport before arrival. For the remaining 424 children, detailed follow-up information was unavailable. Reported reasons for non-arrival included admission to another hospital, parental refusal of transfer, clinical improvement leading to cancellation of referral, cancellation by the referring hospital, or unavailable follow-up information. Among the 334 children admitted to the PICU, 85 died, resulting in a PICU mortality of 25.4%. An additional 15 children died after discharge from the PICU while still hospitalized. These deaths were not included in the primary outcome analysis because the pre-defined primary outcome was PICU mortality. During the study period, four interruptions of SISRUTE occurred, with the longest system outage lasting two weeks.

The baseline demographic, clinical, and referral characteristics of survivors and non-survivors are presented in Table 1. The most common emergency assessment at the referring hospital, based on the PAT, was CNS dysfunction. The most frequently administered pre-referral treatments were oxygen therapy, antibiotic administration, and fluid resuscitation. Compared with survivors, non-survivors had significantly higher PELOD-2 scores and were more likely to require intubation, mechanical ventilation, and vasoactive support before referral.

**Table 1:** Baseline characteristics related to primary outcome.

*Characteristic*	All	Non-Survivor	Survivor	*P-value*
	*N = 334*	*N = 85*	*N = 249*	
Age, month [IQR]	72 [12;144]	36 [12;144]	72 [12;144]	0.185
Gender, Male, n (%)	181 (54.2)	46 (54.1)	135 (54.2)	1.000
Malnutrition, n (%)	176 (52.7)	45 (52.9)	131 (52.6)	1.000
Referral Distance, km [IQR]	48.5 [17.2;101]	65.2 [13.2;180]	46.6 [21.5;100]	0.593
Decision time, min [IQR]	31.5 [13.0;80.0]	38.0 [13.0;67.0]	30.0 [13.0;85.0]	0.798
Emergency category at referring hospital, n (%)				
Respiratory distress/failure	70 (22.9%)	19 (25.3%)	51 (22.1%)	
Circulatory failure	17 (5.56%)	7 (9.33%)	10 (4.33%)	
CNS dysfunction	100 (32.7%)	16 (21.3%)	84 (36.4%)	
Metabolic condition	8 (2.61%)	2 (2.67%)	6 (2.60%)	
Other	46 (15.0%)	11 (14.7%)	35 (15.2%)	
LOS at referring hospital, day (IQR)	2 [1; 5]	3 [1; 5]	2 [1; 5]	0.874
Treatment prior to referral:				
Oxygen therapy, n (%)	229 (68.6%)	52 (61.2%)	177 (71.1%)	0.118
Intubation and manual PPV, n (%)	88 (26.3%)	36 (42.4%)	52 (20.9%)	<0.001
Mechanical ventilation, n (%)	65 (19.5%)	24 (28.2%)	41 (16.5%)	0.027
Fluid resuscitation, n (%)	138 (41.3%)	37 (43.5%)	101 (40.6%)	0.725
Vasoactive drugs, n (%)	77 (23.1%)	34 (40.0%)	43 (17.3%)	<0.001
Antibiotics, n (%)	248 (74.3%)	70 (82.4%)	178 (71.5%)	0.067
LOS at receiving hospital, day [IQR]	4.00 [2.00;9.00]	5.00 [2.00;11.0]	4.00 [2.00;8.00]	0.482
Total hospital length of stay, day [IQR]	9.00 [5.00;15.0]	5.00 [2.00;11.0]	10.0 [6.00;16.0]	<0.001
Transfer time, min [IQR]	221 [144;360]	233 [148;442]	210 [144;326]	0.186
PELOD-2 Score at referral PICU admission [IQR]	3.00 [1.00;6.00]	6.00 [2.00;11.0]	2.00 [1.00;5.00]	<0.001
				

IQR = Inter-Quartil Range, CNS = Central Nervous System, LOS = Length of Stay, PPV = Positive Pressure Ventilation, PELOD-2 score = Pediatric Logistic Organ Dysfunction

No evidence of problematic multicollinearity was identified among the candidate variables included in the multivariable model. All VIF values were below 2.0.

The results of the univariable and multivariable logistic regression analyses are presented in Table 2. In the multivariable model, pre-referral vasoactive support remained independently associated with PICU mortality (adjusted OR 2.23, 95% CI 1.20–4.15; P = 0.011). Furthermore, each 1-point increase in the PELOD-2 score was independently associated with a 32% increase in the odds of PICU mortality (adjusted OR 1.32, 95% CI 1.20–1.45; P < 0.001). Referral distance, decision time, transfer time, age, sex, nutritional status, and pre-referral intubation were not independently associated with PICU mortality after multivariable adjustment.

**Table 2:** Univariable and multivariable logistic regression analyses of factors associated with PICU mortality.

Variable	cOR (95%CI)	*P -*value	aOR (95% CI)	*P -*value
Age (per month)	0.997 (0.994–1.001)	0.171	1.00 (0.99–1.00)	0.330
Male sex	1.00 (0.61–1.63)	0.987	0.99 (0.56–1.74)	0.970
Malnutrition	0.99 (0.60–1.62)	0.958	0.79 (0.44–1.41)	0.424
Referral distance (per 10 km)	1.02 (0.99–1.05)	0.156	1.02 (0.98–1.07)	0.295
Decision time (per 10 min)	1.00 (0.98–1.02)	0.791	0.99 (0.97–1.02)	0.620
Transfer time (per 60 min)	0.85 (0.68–1.05)	0.132	1.00 (0.72–1.40)	0.997
Intubation prior referral	**2.78 (1.64–4.72)**	**<0.001**	0.59 (0.28–1.25)	0.168
Vasoactive support prior referral	**3.19 (1.85–5.50)**	**<0.001**	**2.23 (1.20–4.15)**	**0.011**
PELOD-2 score (per 1-point increase)	1.30 (1.21–1.40)	**<0.001**	**1.32 (1.20–1.45)**	**<0.001**

cOR, crude odds ratio; aOR, adjusted odss ratio; CI, confidence interval; PELOD-2, Pediatric Logistic Organ Dysfunction-2

## Discussion

To our knowledge, this is among the first prospective studies to investigate factors associated with PICU mortality among critically ill children transferred and referred to a tertiary care PICU in Indonesia. The main findings of this study were that the need for pre-referral vasoactive support was independently associated with PICU mortality. In contrast, referral distance, decision time, transfer time, age, sex, nutritional status, and the need for pre-referral intubation were not independently associated with mortality after multivariable adjustment. This study also demonstrated that higher PELOD-2 scores measured within the first 24 hours after PICU admission were independently associated with PICU mortality. Each one-point increase in the PELOD-2 score was associated with a 32% increase in the odds of PICU mortality.

In this study, only approximately one-third of children referred for PICU admission were ultimately admitted to the PICU. This finding is consistent with our previous study [3]. The outcomes of children who never reached our hospital remain largely unknown because detailed follow-up information was unavailable. Ineffective and delayed communication between referring and receiving tertiary-level hospitals may contribute to this problem. The communication system currently used is a web-based application created by the Ministry of Health (SISRUTE). Although initially this system was designed to enable prompt information exchange with the aim of improving patient safety, several operational limitations remain. The system lacks a dedicated mechanism to prioritize and handle urgent cases requiring immediate action. It does not include real-time notifications for incoming messages; instead, the referring hospital must manually refresh the application to check for responses [3,14]. Not all hospitals have designated personnel responsible for monitoring the application. In addition, a referring hospital can send referral requests to multiple hospitals. The patient will be transferred to the hospital that first accepts the referral request, even though the accepting hospital may not have the appropriate resources to manage the patient, and there is rarely any feedback to us if the patient has been accepted at another hospital. The ability to send referral requests to multiple hospitals without a structured feedback mechanism may contribute to non-arrival; however, the absence of detailed follow-up data for most non-arriving patients prevents us from determining the magnitude of this effect. This represents an important challenge in the current referral system.

Our findings should not be interpreted as evidence that expanding PICU capacity or upgrading healthcare infrastructure would directly reduce mortality. However, together with previous literature describing referral-system challenges in LMICs, these findings highlight areas that warrant further evaluation through implementation and health-system research. There is a need for standardized e-referral slips tailor-made to each state of the patient with contextualized protocols for early recognition of complications and effective communication between health care facilities [19–21]. The establishment of an e-referral system for critically ill children necessitates collaboration from multiple stakeholders, alongside adequate facilities and infrastructure across all levels of healthcare services to ensure the system’s objectives are effectively achieved.

As only 31% of the referred and arrived patients to our hospital needed PICU admission, the role of GPs working in referring hospitals also warrants strengthening. Strengthening the knowledge, clinical skills, and training of general practitioners in recognizing critically ill children, identifying indications for PICU referral, and providing early stabilization before transfer may improve referral appropriateness and optimize the use of limited PICU resources. Although this hypothesis is biologically and operationally plausible, our observational design does not allow us to determine whether these interventions would improve mortality.

The need for pre-referral vasoactive support was independently associated with PICU mortality in the present study. This finding likely reflects greater illness severity, particularly circulatory failure or shock requiring pharmacological cardiovascular support before transfer. While evidence from adult populations suggests that inotropic and/or vasopressor use are strong predictors of in-ICU death [21], the evidence in pediatric populations is more complex [22–24]. A retrospective cohort study demonstrated that the need for vasoactive drugs was independently associated with increased mortality in children with septic shock, even after adjustment for illness severity and other important confounders, including PRISM III score, lactate concentration, age, corticosteroid use, blood transfusion, and fluid resuscitation [25].

Shock severity is traditionally defined by the presence of hypotension; however, elevated blood lactate, metabolic acidosis, prolonged capillary refill time, and other indicators of tissue hypoperfusion may also reflect circulatory compromise [26]. Unfortunately, many of these parameters are not routinely available at referring hospitals in our setting, potentially delaying recognition and appropriate stabilization before transfer. Furthermore, documentation of shock management at referring hospitals was frequently incomplete, and communication between healthcare providers during referral and transport was often inadequate. Unlike pre-referral intubation, which was not independently associated with mortality after multivariable adjustment, the need for vasoactive support remained significant. This finding suggests that persistent circulatory instability before transfer may provide prognostic information beyond the severity of illness measured once the patient is admitted in the PICU. This observed association may also be influenced by the underlying etiology of shock. Our data did not allow reliable differentiation between septic, cardiogenic, hypovolemic, and other forms of shock, which may have different prognoses independent of vasoactive use

In the present study, higher PELOD-2 scores measured within the first 24 hours after PICU admission were independently associated with PICU mortality. Specifically, each one-point increase in the PELOD-2 score was associated with a 32% increase in the odds of PICU mortality. Several validated scoring systems have been developed to assess illness severity and predict mortality in critically ill children, including the (PELOD) score and the Pediatric Risk of Mortality (PRISM) score, or the Pediatric Index of Mortality (PIM) score. PELOD-2 evaluates dysfunction across multiple organ systems, including the cardiovascular, respiratory, neurological, hematological, renal, and hepatic systems, and is routinely used in our PICU. Previous studies from Indonesia have evaluated the predictive performance of PELOD-2 using data-driven cut-off values. One study reported that a PELOD-2 cut-off >4.5 demonstrated good discrimination for mortality, whereas another study using a modified PELOD-2 score identified an optimal cut-off of 6.5 [27–29]. However, unlike other studies that focused on identifying optimal cut-off values, we analysed PELOD-2 as a continuous variable to preserve statistical information and avoid the potential bias associated with categorising continuous predictors. Therefore, our findings should be interpreted as demonstrating that increasing illness severity after PICU admission is associated with mortality, rather than supporting a specific PELOD-2 threshold for clinical decision-making. Because PELOD-2 was assessed within the first 24 hours after PICU admission, it should be interpreted as an admission severity marker rather than a pre-referral predictor. Illness severity at PICU admission may have been influenced by pre-referral stabilization, transport, and clinical deterioration before arrival.

Given the high PICU mortality among children successfully transferred to the tertiary PICU, together with the number of children who died before reaching the referral center, the referral system for critically ill children in our setting requires substantial improvement. Although referral distance, decision time, and transfer time were not independently associated with PICU mortality, these findings should not be interpreted as evidence that referral processes are unimportant. Rather, they likely reflect the complex interplay between illness severity, stabilization before transfer, transport quality, and communication between healthcare facilities. Previous studies have suggested that direct, real-time communication may facilitate interfacility referrals for critically ill children; whether similar approaches improve outcomes in our setting requires prospective evaluation [16,29,30]. Strengthening healthcare infrastructure should include the development of dedicated pediatric transport teams, expansion of PICU capacity at regional hospitals, and enhanced training of GPs in the early recognition, stabilization, and timely referral of critically ill children.

### Study limitations

This study has several limitations. First, detailed information regarding pre-referral stabilization was frequently unavailable or incompletely documented. Variables such as fluid resuscitation volume, vasoactive drug dosage, blood lactate concentration, base deficit, shock classification, and airway management expertise could therefore not be incorporated into the analysis, potentially resulting in residual confounding. Intermittent interruptions of the SISRUTE system may also have contributed to incomplete referral documentation and information bias. In addition, shock etiology could not be reliably classified; therefore, residual confounding related to differences in shock type and severity cannot be excluded.

Second, this study included only children who were successfully transferred and admitted to the PICU. Eleven children who arrived at the receiving hospital but died in the ED before PICU admission did not meet the prespecified inclusion criteria, and the individual-level predictor data required for the planned analysis were therefore not prospectively collected. Consequently, a sensitivity analysis including these patients could not be reliably performed. Their exclusion may have introduced selection bias, and our findings should be interpreted as factors associated with PICU mortality among children who survived to PICU admission, rather than mortality across the entire referral pathway.

Third, although all eligible patients during the study period were included, the relatively limited number of outcome events may have affected model stability and restricted the number of predictors that could be included in the multivariable analysis. Referral distance was estimated using Google Maps rather than actual ambulance travel distance, which may have introduced measurement error. Future multicenter studies with larger sample sizes and complete referral pathway data are warranted to externally validate these findings.

Finally, this was a single-center study conducted on the island of Java. Referral geography, transportation networks, healthcare infrastructure, and access to pediatric critical care differ substantially across Indonesia; therefore, these findings may not be generalizable to settings in other islands, including Sulawesi, Kalimantan, Maluku, and Papua.

## Conclusion

In conclusion, critically ill children transferred and admitted to a tertiary-care PICU in Indonesia experienced substantial PICU mortality. Pre-referral vasoactive support and higher PELOD-2 scores at PICU admission were independently associated with mortality. These findings suggest that early stabilization before transfer and communication during interhospital referral deserve further evaluation as potential targets for quality-improvement initiatives and implementation research.
